# Prevalence of tanning equipment use among Canadians

**DOI:** 10.1016/j.pmedr.2021.101356

**Published:** 2021-03-11

**Authors:** Sami S. Qutob, James P. McNamee, Orly Brion

**Affiliations:** a Consumer and Clinical Radiation Protection Bureau, Health Effects and Assessment Division at Health Canada, Canada; b Environmental Health Science and Research Bureau, Population Studies Division at Health Canada, Canada

**Keywords:** Eye protection, Injury, Sunburn, Sunlamp, Survey, Tanning equipment, Ultraviolet radiation, Warning labels, Tanning bed, Sunbed, and Indoor tanning

## Abstract

The objective of this study was to collect prevalence estimates of indoor tanning usage and associated injuries in Canada. The rapid response component of the 2019 Canadian Community Health Survey collected data on the use of tanning equipment in the previous 12 months, including reasons for use, frequency/duration of use, precautions taken and adverse reactions or injuries.

The 2019 research findings were as follows, an estimated 3.0% (95% CI: 2.5–3.4%) of Canadians reported that they had used indoor tanning equipment in the past year. Among users, 71.1% (95% CI: 63.9–78.3%) were female and females aged 18–34 were significantly more prevalent users compared to females aged 45 or older. The prevalence of indoor tanning was higher among people without a university degree; however, there were no differences in prevalence by household income or region. Most users indicated they used indoor tanning equipment within a tanning salon (75.3%: 95% CI: 69.1–81.6%) and the most common reason for usage was to develop a “protective” base tan (72.1%: 95% CI: 65.2–78.9%). Over one third (39.2%: 95% CI: 31.1–47.2%) of all users reported 10 or more sessions in the past year.

The prevalence of indoor UV tanning usage is declining in Canada. Similar to results in 2014, the majority of users continue to be female, with a large number in the 18 to 34 age group.

## Highlights

• In 2019, an estimated 3% of Canadians aged 12 or older used indoor UV tanning.
• Indoor tanning remained most prevalent among women, particularly at ages 18 to 34.
• Development of a base tan is the primary motivation for using tanning equipment.
• Most users have read the warnings labels at least once when “read them in the past” is considered.

## 1 Introduction

Skin cancer continues to be the most common type of cancer in Canada (Canadian Cancer Society, 2019). Non-melanoma skin cancers, such as squamous and basal cell carcinomas, are most common, but rarely fatal, and in most cases, caused by ultraviolet (UV) radiation exposure. Melanoma is a less common, but potentially deadly form of skin cancer. It has been estimated that 62–65% of melanoma cases have been attributed to UV radiation (Kim and He, 2014, O'Sullivan et al., 2019). In 2019, it was estimated that 7,800 Canadians would develop melanoma and that 1,300 would die from it (Canadian Cancer Society, 2019). Its southern neighbour, the Unites States, has attributed 9000 deaths to melanoma, in 2018 (Bowers et al., 2020). Cases of melanoma continue to rise with incidence rates increasing by over 50% in both Canadian males and females over the last three decades, a possible consequence of delayed emergence of the disease from unprotected exposures to UV early in life that is overrepresented in an aging population (O'Sullivan et al., 2019, Xie et al., 2015).

An increased risk of skin cancer, sunburn, premature skin aging, immunosuppression, eye diseases, such as cataracts, have all been associated with prolonged and repeated exposure to UV radiation from unprotected natural tanning outdoors and/or using tanning equipment to develop a tan (Xie et al., 2015, Gallagher and Lee, 2006). Based upon the weight of scientific evidence, the use of UV-emitting tanning devices was classified as “carcinogenic to humans” (Group 1), in 2009, by the International Agency for Research on Cancer (IARC) and the risk of developing skin melanoma has been estimated to increase by 59% when tanning equipment use begins before age 35 (El Ghissassi et al., 2009, International Agency of Research on Cancer, 2007, Boniol et al., 2012). Repeated or prolonged use of tanning equipment only increases this risk (Lazovich et al., 2010). In Canada, the combination of sunburn, intentional sunbathing, and indoor tanning has been estimated to account for more than a quarter of all melanomas, with sunburn, sunbathing and indoor tanning accounting for 7.4%, 17.8%, and 7.0%, respectively (O'Sullivan et al., 2019). Furthermore, it has been estimated that non-melanoma skin cancers attributable to indoor tanning are responsible for 5.2% of basal cell and 7.5% of squamous cell carcinomas in Canada (O'Sullivan et al., 2019).

Similar to 2014, the 2019 Canadian Community Health Survey (CCHS) collected information from residents of the 10 provinces on tanning equipment use in the previous year. The data include the prevalence and frequency of use and reports of injuries or discomfort to either the eyes or skin, reasons for use, location of use, and the efficacy of labelling and safety information provided with these devices. Documented changes in the prevalence estimates of tanning equipment use and associated injury can assist in monitoring current and predicting future impacts on public health.

## 2 Data and methods

### 2.1 Data source

Statistics Canada’s CCHS collects health-related data from the Canadian population for use at the national, provincial and regional levels. The data analyzed in this article are from the CCHS rapid response module on Tanning Equipment Use, which took place from March through June 2019. Respondents were asked about their use of tanning equipment during the previous 12 months.

The CCHS questionnaire was administered directly by phone to respondents using computer-assisted telephone interviewing. The rapid response module of the CCHS covers the household population aged 12 or older in all provinces, excluding the three territories. The survey also excludes people living on reserves and other Aboriginal settlements in the provinces; full-time members of the Canadian Forces; residents of institutions; and residents of the Quebec health regions of Région du Nunavik and Région des Terres-Cries-de-la-Baie-James. Together, these exclusions represent <3% of the population aged 12 or older. Overall, 22,527 individuals were in-scope for the 2019 CCHS module on tanning equipment usage, with responses obtained from 12,397 individuals (5,665 males and 6,732 females), yielding a response rate of 55.0%. A detailed description of the CCHS methodology and sources used can be found on the Statistics Canada website: https://www23.statcan.gc.ca/imdb/p3Instr.pl?Function=assembleInstr&lang=en&Item_Id=1232710.

## 3 Measures

Much like 2014, the 2019 CCHS rapid response survey tanning module was divided into different sets of questions. Based on responses from the 2014 survey, some questions were modified and re-phrased for additional clarity. The first set identified vulnerable populations, based on characteristics affecting the prevalence of use, who may be at higher risk for injury; for example, “In general, when you're outside, how does your skin react to unprotected exposure to the sun?”. As a screener question, all respondents were asked “Have you ever used tanning equipment?”. The second set of questions asked about the frequency of and reasons for the use of sunlamps or tanning equipment in the past year. Use and prevalence of injury or adverse reactions to the skin and eyes were also asked of the respondents and whether injury to the skin required treatment by a health care professional. Whereas, other questions addressed precautions taken by users; for example, “Did you wear tanning goggles to protect your eyes?”

In Canada, the Radiation Emitting Devices Regulations, Schedule II, Part XI, (amended 2018–12-29) require that all tanning equipment imported, sold, leased or advertised in Canada have a permanently affixed warning label and information regarding the recommended exposure time for each tanning session based on the individual’s skin type. As in the 2014 survey, some questions asked if users were aware of the device warning labels and read the instructions posted on the equipment, and if not, why.

Finally, questions were asked about the location of use. All questions are available on the Statistics Canada website: https://www23.statcan.gc.ca/imdb/p3Instr.pl?Function=assembleInstr&lang=en&Item_Id=1232710.

### 3.1 Statistical analysis

The analyses were based on a sample of 12,397 respondents, aged 12 or older, living in the 10 provinces. To be representative of the Canadian population, data analyses were weighted and carried out using SAS EG 7.1 (SAS Institute Inc., USA). The SAS procedure SURVEYFREQ was used to estimate percentages and coefficients of variation (CVs). Estimates with a CV from 16.6% to 33.3% are identified by an (E) and should be interpreted with caution; estimates with a CV greater than 33.3% are suppressed (F) due to extreme sampling variability. To test differences in prevalence between sociodemographic groups, the procedure SURVEYLOGISTIC was used to calculate odds ratios and corresponding confidence intervals with Bonferroni adjustments for pairwise comparisons. Both procedures (SURVEYFREQ and SURVEYLOGISTIC) took account of sampling weights and estimated variance using bootstrap weights. Differences in estimates from different cycles of CCHS (2019 versus 2014) were tested using a 2-sided Z-test. Note: Throughout the text all values in the parentheses represent 95% confidence intervals if not stated within the provided tables.

## 4 Results

### 4.1 Prevalence of use based age and sex

An estimated 3% (2.5–3.4%) of Canadians (940,317 (788,581–1,092,052) individuals) used indoor UV tanning equipment in the past year (Table 1). The females’ odds of using tanning equipment were significantly higher than males’ (OR: 2.5 (1.7–3.5), p < 0.0001) (Table 1). Canadians aged 18 to 34 years had the highest prevalence of use, followed closely by those aged 35 to 44 years, which was more prominently reflected amongst the female population (Table 1). There were an insufficient number of respondents under age 18 to yield reportable estimates.

**Table 1:** Prevalence of and unadjusted odds ratios relating tanning equipment use in past year to selected characteristics, household population aged 12 or older, Canada excluding territories, 2019.

Characteristic	%	95% confidence interval		Odds ratio	95% confidence interval	
		from	to		from	to
**Total population**						
Used tanning equipment (last 12 months)	3.0	2.5	3.4			
**Sex**						
Male†	1.7	1.2	2.2	1.00	…	…
Female	4.2	3.3	5.0	2.46*	1.72	3.51
**Age group**						
12 to 17	…^F^	…	…	…^F^	…	…
18 to 34	4.5	3.4	5.6	2.17*	1.39	3.39
35 to 44	4.3^E^	2.5	6.1	2.06*	1.11	3.85
45 or older†	2.1	1.6	2.6	1.00	…	…
**Female age group**						
12 to 17	…^F^	…	…	…^F^	…	…
18 to 34	6.9	5.1	8.7	2.53*	1.50	4.28
35 to 44	5.7^E^	2.5	8.9	2.08	0.87	4.97
45 or older†	2.8	2.0	3.7	1.00	…	…
**Education**						
Secondary diploma or equivalent or less	2.1	1.6	2.6	1.45	0.87	2.42
Some postsecondary (certificate/diploma including trade)	5.8	4.4	7.2	4.19*	2.48	7.09
University certificate, diploma or degree†	1.5^E^	0.9	2.0	1.00	…	…
**Household income**						
$39,999 or less†	2.8^E^	1.8	3.9	1.00	…	…
$40,000 to $69,999	2.1^E^	1.3	3.0	0.75	0.38	1.48
$70,000 to $99,999	2.5^E^	1.7	3.2	0.87	0.45	1.67
$100,000 to $149,999	3.9^E^	2.6	5.1	1.38	0.71	2.68
$150,000 or more	3.4^E^	2.2	4.5	1.20	0.62	2.35
**Region**						
Atlantic	3.6^E^	2.1	5.2	1.52	0.71	3.25
Quebec	3.5	2.5	4.5	1.46	0.84	2.55
Ontario†	2.4^E^	1.5	3.3	1.00	…	…
West	3.1	2.4	3.9	1.30	0.76	2.25
**Race (Ethnicity)**						
Caucasian	3.8	3.2	4.4	12.12*	5.04	29.19
Other†	…^F^	…	…	1.00	…	…

… not applicable.

**Source:** 2019 Canadian Community Health Survey.

* Significantly different from reference category (p < 0.05).

F unreliable to publish.

E use with caution. Coefficient of variation (CV) between 16.6% and 33.3%.

† reference category.

The majority of users were female 71.1% (63.9–78.3%), with a higher prevalence of use among women under age 45. Females aged 18–34 accounted for 42.4% (33.1–51.8%) of female users while females aged 35–44 accounted for 21.6%^E^ (11.5–31.8%) of female users. Males aged 18–34 accounted for 34.5%^E^ (20.4–48.6%) of all male users with the majority of male users in the 45 + age category (40.0% (27.0–53.0%). Age differences were found to be significant as well: prevalence of indoor tanning was significantly higher in both the 18–34 and 35–44 age groups, compared to the 45 + age group (ORs: 2.2 (1.4–3.4); and 2.1 (1.1–3.9), respectively) (Table 1).

### 4.2 Prevalence of use based on education, household income and region

The prevalence of tanning equipment use was higher among people with “some postsecondary (certificate/diploma),” compared with among those with a “university certificate, diploma or degree” and of those with “secondary graduation or less”. The prevalence among people with some postsecondary education was significantly higher than among people with a university degree (OR:4.2 (2.5–7.1). The prevalence among people with a secondary diploma (or less) was also higher than among people with a university degree (OR:1.5 (0.9–2.4), but not significantly. No statistically significant differences existed in the prevalence of indoor UV tanning across income levels. Prevalence of tanning equipment usage across regions was the lowest in Ontario and the highest in the Atlantic region, however there were no significantly different regional differences (Table 1).

### 4.3 Frequency and location of tanning equipment use and reasons for use

Among tanning equipment users, 13.0%^E^ considered themselves to have sunburn-sensitive skin (“Always burns, never tans” and “Usually burns, tans minimally”, skin type I and II) (Table 2), placing them at higher risk for UV-related skin cancer. When frequency of tanning equipment usage among users was assessed, the majority reported 10 sessions or more in the past year. The prevalence of users reporting 3 sessions or less were similar to those reporting 4–6 sessions. Users were more likely to use tanning equipment in a tanning salon, followed by fitness centres/health clubs and beauty salons/hair dresser/spa (Table 2). The leading reason for indoor UV tanning was to develop a base tan followed by aesthetic reasons, and then to relax or feel better. Far fewer users reported reasons such as to obtain vitamin D, to boost the immune system or other reasons (Table 2).

**Table 2:** Factors involved in tanning equipment use, household population aged 12 or older, Canada excluding territories, 2019.

Factor	% of users	95% confidence interval	
		from	to
**Place of use**			
Beauty salon / hair dresser / spa	5.8^E^	3.5	8.2
Tanning salon	75.3	69.1	81.6
Fitness centre / health club	15.6^E^	10.0	21.3
Private home (your home or someone else's)	…^F^	…	…
Another place	…^F^	…	…
**Reasons for use^§^**			
Get base tan	72.1	65.2	78.9
Aesthetic	31.3	24.6	38.1
Relax/Feel better	24.6	17.5	31.7
To get Vitamin D	15.2^E^	9.9	20.5
Boost immune system	5.8^E^	2.7	9.0
Other	7.8^E^	4.5	11.2
**Frequency of use (per year)**			
1–3 times	25.8	19.0	32.6
4–6 times	22.5^E^	15.7	29.3
7–9 times	11.6^E^	6.8	16.5
10 + times	39.2	31.1	47.2
**Wore eye protection**			
Always (Yes)	78.2	71.2	85.1
Never (No)	11.8^E^	7.2	16.5
Sometimes	…^F^	…	…
Often	…^F^	…	…
**Read warning labels each session in past year**			
Yes	43.6	35.9	51.3
No	56.4	48.7	64.1
**Reasons for not reading warning labels**			
Read in past	73.8	64.1	83.5
Never read	21.8^E^	12.3	31.4
Did not notice labels/none posted/illegible	…^F^	…	…
**Read manufacturer's instructions in past year**			
Yes	24.0	17.6	30.4
No	74.3	67.4	81.1
**Reasons for not reading manufacturer's instructions**			
Read in the past	58.3	48.9	67.7
Never read	23.8^E^	16.7	30.8
Did not notice labels/none posted/illegible	17.9^E^	9.3	26.6
**Determining time of session**			
Someone else (not user) determined the time	45.1	36.9	53.3
Used manufacturer's recommendations	10.7^E^	5.9	15.6
Self-determined the time	41.0	33.3	48.7
Another way	…^F^	…	…
**Skin reaction to unprotected exposure to the sun**			
Always burns, never tans	…^F^	…	…
Usually burns, tans minimally	13.0^E^	8.3	17.6
May burn, tans well	40.4	32.6	48.3
Rarely burns, tans well	44.1	36.1	52.0
**Discomfort or unwanted reaction to skin in past year**			
Often	…^F^	…	…
Occasionally	3.2^E^	1.4	5.0
Once	4.6^E^	2.0	7.1
Never	92.2	89.0	95.4

… not applicable.

§ respondents could report more than one reason.

**Source:** 2019 Canadian Community Health Survey.

F unreliable to publish.

E use with caution. Coefficient of variation (CV) between 16.6% and 33.3%.

### 4.4 Warning labels and manufacturer’s instructions

Less than half (43.6%) of users read the warning labels on the tanning equipment during each session. Among the 56.4% who did not, the most common reason was “have read them in the past” followed by “I have never read them”. These findings suggest that 85.2% (79.4–91.0%) of respondents have read the labels at least once when you include those who have not read the labels in the last session as they have read them before. The majority of users also did not read the manufacturer's recommendations for determining the duration of each tanning session. The main reasons for not reading the manufacturer's instructions were “they read it in the past” followed by “never read them”. The duration of each session was usually determined by someone else or was self-determined by the user, with only 10.7%^E^ using the manufacturer's instructions.

### 4.5 Eye protection and eye/skin discomfort or injury reporting

Most users wore eye protection during their tanning sessions and there were few reports of eye discomfort or injury (estimates not shown). Skin reaction/discomfort was reported to occur occasionally (3.2%^E^) or only once in the past one-year time period (4.6%^E^). Reports of skin injury requiring treatment from a health care professional were too infrequent to publish.

## 5 Discussion

**Fig. 1:**
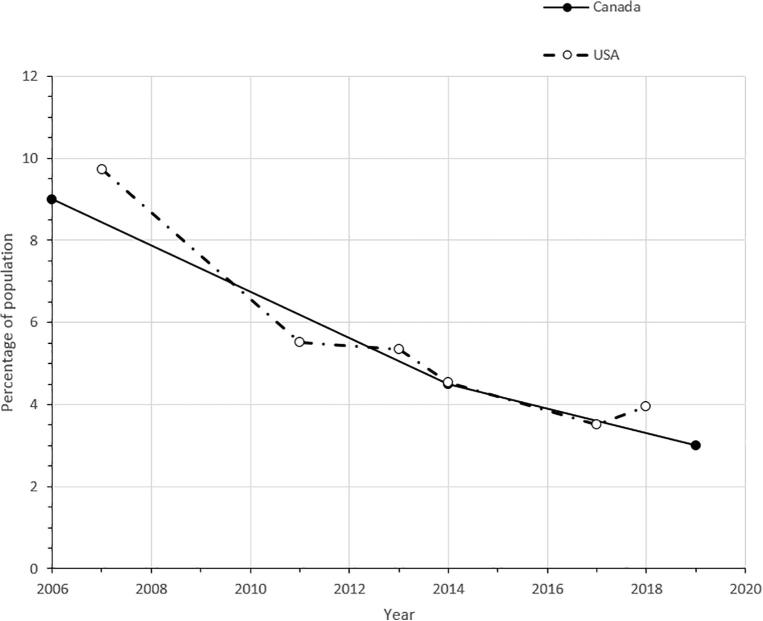
Past-Year Indoor Tanning Prevalence in Canada and the United States. **Sources:** 2006 Second National Sun Survey, 2014 and 2019 Canadian Community Health Survey. Health Information National Trends Survey (HINTS), United States, 2007–2018 **NOTE:** Confidence intervals were not presented as they were not available for all the data points, particularly from the US study.

The objective of this study was to not only capture current prevalence estimates of indoor tanning usage and associated injuries in Canada but to document any changes in those prevalence estimates from earlier studies. The data seems to suggest that the prevalence of indoor UV tanning usage is declining in Canada. In 2006, the National Sun Survey (NSS) collected data on sun exposure and tanning behaviour from people aged 16 or older across Canada, reporting that 9% of Canadians had used indoor UV tanning equipment during the previous 12 months (Marrett et al., 2010, Ontario Sun Safety Working Group, 2006). In 2014, Health Canada, through the CCHS, reported that the prevalence of self-reported tanning equipment usage in the previous 12 months had dropped to 4.5% (4.1–4.9%) for Canadians aged 12 or older (Qutob et al., 2017). Based on data from CCHS 2019, the estimated prevalence in tanning equipment usage has further dropped in 2019, to 3.0% (2.5–3.4%). This represents a statistically significant change (p < 0.0001) from the 2014′s estimate. The 2019s estimate (3%) is comparable to the United States’ 2018 indoor tanning prevalence estimate of 4%; to which the US has shown a 6% decrease from 11 years earlier (Bowers et al., 2020). Taken together, these study findings show a similar 6% decline of tanning equipment use in North America over the last 13 years (Fig. 1). The downward trend of tanning equipment use in Canada may be attributable to changes in provincial legislation regulating the sale and marketing of tanning services to youth, stronger federal health warning labels and a greater understanding among the population of the risks associated with intentional tanning (Nadalin et al., 2018). The IARC classification of UV radiation from tanning equipment as “carcinogenic to humans” has been a driving force in increasing the publics’ awareness of risk from UV tanning and for prompting policy changes in multiple jurisdictions (International Agency for Research on Cancer, 2012, Rodriguez-Acevedo et al., 2020).

Similar to both the 2014 CCHS and the 2006 NSS, findings from the current analysis indicate that indoor UV tanning was more prevalent among young females. The finding is similar despite limitations related to CCHS redesign in 2015 (Statistics Canada’s Canadian Community Health Survey (CCHS), 2020). This finding is consistent with other studies which have identified females as being a higher user group placing them at increased risk (Wehner et al., 2014). According to the current study, 4.2% of women used tanning equipment, compared with 1.7% of men. The 2014 CCHS found 6.2% usage among women and 2.7% among men, while in the 2006 NSS, 11% of women used tanning equipment versus 3% of men (Marrett et al., 2010, Qutob et al., 2017). Several studies have also reported that indoor tanning use is more common among younger women than among those aged 45 or older (Marrett et al., 2010, Qutob et al., 2017, Lim et al., 2005, Heckman et al., 2008). A recent study reported that certain sexual and gender minorities have an increased prevalence of indoor tanning usage (Marks et al., 2020); however, the current study was not able to confirm these findings, in regards to gender identification, due to an insufficient number of responses from respondents.

A new question added to the current study CCHS concerned the location of where the tanning equipment was used as it has been suggested that usage within private residences may raise the risk for adverse health effects due to operation by untrained users (Marks et al., 2020). The current study found that the majority of use was within tanning salons (an estimated 708,435 (571,296 to 845,573) Canadian users), followed by fitness centres/health clubs and then beauty salons/hair dressers/spas and very few respondents declared using tanning equipment in private homes. The results of this study are consistent with others that have reported that the majority of use still occurs primarily in commercial tanning facilities where it is assumed that exposure is controlled by knowledgeable operators (Rhainds et al., 1999, Gavin et al., 2010, Ferrucci et al., 2014, Nadalin et al., 2016). However, previous research has reported that risk information may often be withheld by tanning facility operators either from a lack of knowledge or from the fear of losing income (Reimann et al., 2019). Other studies have reported that operators of tanning salons often ignore manufacturer’s recommended exposure schedules thereby reducing the potential user protection afforded by these operators (Kwon et al., 2002, Culley et al., 2001). This may be reflected in the 13.0%^E^ of tanning equipment users that have identified themselves as having sunburn-sensitive skin, but continue to use tanning equipment placing them at an increased risk for skin cancer.

Awareness of signage (warning labels and exposure schedule/manufacturer’s instructions) were examined in both the current study and the 2014 CCHS survey (Qutob et al., 2017). Although the scope was somewhat different between the two surveys. The 2019 questions on whether the respondent read the signage were based on the last time that the tanning equipment was used, and the 2014 questions were about reading the signage each time the respondent used the equipment. Despite this difference, the prevalence for reading warning labels was similar, with the 2019 study reporting a lower prevalence of respondents that read them (2014: 51.7% (46.8–56.5%) vs 2019: 43.6% (35.9–51.3%) (p = 0.0808)). The majority of users have read the labels as the prevalence of reading it at least once (reading plus not reading due to reading in the past) was found to be 81.2% (77.3–85.0%) in 2014 and 85.2% (79.4–91.0%) in 2019. In 2019, the prevalence for reading the manufacturer’s recommendation on the duration of each tanning session was only 24.0% compared to 80.2% in 2014. The large difference between these estimates may have been due to how the questions were phrased between the two surveys (e.g. ‘reading’ vs ‘following’). The reasons for responding negative to either question were most frequently reported as having read or followed the posted literature sometime in the past. Regarding reading manufacturer’s instructions for use, a lack of clearly posted labels/information was reported by some users.

Motivation for use of tanning equipment was also examined. The motivation of 72.1% of tanning equipment users was to protect against future sunburns or to develop a base tan. This is relatively unchanged relative to the findings from the 2006 NSS where 74% of users reported they used tanning equipment to get a base tan, but up slightly from the 2014 CCHS at 62.0% (Marrett et al., 2010, Qutob et al., 2017). Despite changes in the overall prevalence of indoor tanning equipment usage, the motivation among users is still guided by misinformation as a base tan provides little protection against sunburns, with no evidence of reducing the risk of developing melanoma or non-melanoma skin cancers (Miyamura et al., 2011, Bech-Thomsen and Wulf, 1996, Hollovary, 2009, Eller and Gilchrest, 2000, Lim et al., 2011). Differences in prevalence between previous and the current studies may not only be related to a change in behaviour as a result of understanding the risk. It may also be a result of a shift in public opinion about what is socially acceptable from the increased presence of information campaigns on the health risks of tanning. Lastly, it would had been of some interest to examine the distribution of misinformation (e.g. to “boost immune response”) among the different age and education categories however this analysis could not be done due to the low prevalence and large uncertainty associated with this data.

Fortunately, there was a low prevalence of acute injuries from tanning equipment among users. The prevalence for skin discomfort or unwanted reaction in the previous year among users was 7.8%^E^ (4.6–11.0%). The combined prevalence for skin discomfort and injury requiring treatment was 11.7%^E^ (5.9–17.5%) similar to the 10.4% (7.5–13.2%) of users reporting discomfort or injury in 2014 (Qutob et al., 2017). The current study found 11.8% ^E^ of tanning equipment users did not wear eye protection which is similar to a prevalence of 14.0% of users who did not wear eye protection in 2014. Other studies have reported a higher prevalence of tanning equipment users not wearing protective goggles (Bowers et al., 2020, Knight et al., 2002, Szepietowski et al., 2002).

This survey is a 5-year follow-up to the 2014 CCHS analysis of tanning equipment use by Canadians. These findings will allow for an assessment of changes in tanning equipment usage behaviour among Canadians, particularly following recent amendments, in 2018, to tanning equipment labelling requirements under the *Radiation Emitting Devices Act* (Department of Justice Canada, 2020) and provincial age restrictions on tanning equipment use by those under 18 years of age. The decreased prevalence rate of tanning equipment usage in 2019 may be a reflection of the impact of these regulatory changes, but may also reflect an increased understanding of the health risks posed by indoor tanning amongst the Canadian public. Despite the decline in use there is ongoing need for information regarding the prevalence of usage or exposure among Canadians and the prevalence of injuries from such devices to ensure risks from these products are properly assessed and appropriate risk management strategies are implemented.

## 6 Limitations

The findings of this study have limitations which need to be considered when interpreting the results. Due to the redesign of the CCHS in 2015, sample allocation strategies have been changed, including the selection of new time frames, sample allocations, weighting and estimation methodologies, making direct comparisons between the 2014 and 2019 data difficult to establish any strong inferences (Statistics Canada’s Canadian Community Health Survey (CCHS), 2020). For example, questions regarding reading or following signage were different amongst the surveys. The 2014 questions were about reading the signage each time the respondent used the equipment whereas the 2019 questions asked if respondents read them the last time the equipment was used. Also of note, the US HINTS survey is limited to adults (aged 18 and over) so the prevalence of indoor tanning usage may not be directly comparable to the NSS or CCHS survey results since the age cut-off was 16 and 12, respectively.

Similar to the 2014 analysis, the design for some of the questions were not ideal for assessing injuries but were repeated in the current study to allow for comparison to previous results. For example, a single category (discomfort and/or injury) was used to describe a number of types of injury, whether to the eye (itchiness, light aversion, redness) or to the skin (sunburn, discoloration, itch), preventing reporting of specific types of injury to those organs. However, in this study, serious injury to either the skin or eyes that required treatment by a health care professional was infrequently reported.

## 7 Conclusion

Despite a decrease in prevalence over the last decade, almost a million Canadians continue to use tanning equipment. Women, particularly those aged 18 to 34, are at a proportionally higher risk of developing skin cancer from tanning equipment usage due to their increased prevalence of use and their age at use. Among users, the major motivation for use continues to be for the development of a base tan. This reflects an ongoing lack of information and misperception that indoor tanning somehow protects against sunburn from natural outdoor sun exposure.

## CRediT authorship contribution statement

**Sami S. Qutob:** Conceptualization, Methodology, Writing - review & editing. **James P. McNamee:** Conceptualization, Methodology, Writing - review & editing, Project administration, Supervision. **Orly Brion:** Writing - review & editing.

## Declaration of Competing Interest

The authors declare that they have no known competing financial interests or personal relationships that could have appeared to influence the work reported in this paper.
